# Reversal of Autism Symptoms among Dizygotic Twins through a Personalized Lifestyle and Environmental Modification Approach: A Case Report and Review of the Literature

**DOI:** 10.3390/jpm14060641

**Published:** 2024-06-15

**Authors:** Christopher R. D’Adamo, Josephine L. Nelson, Sara N. Miller, Maria Rickert Hong, Elizabeth Lambert, Heather Tallman Ruhm

**Affiliations:** 1 Department of Family and Community Medicine, University of Maryland School of Medicine, Baltimore, MD 21201, USA; saramiller@som.umaryland.edu; 2 Documenting Hope, Windsor, CT 06095, USA; josie@documentinghope.com (J.L.N.); maria@epidemicanswers.org (M.R.H.); beth@epidemicanswers.org (E.L.); drtallmanruhm@documentinghope.com (H.T.R.)

**Keywords:** autism spectrum disorders (ASD), dizygotic twins, total allostatic load, functional medicine, environmental medicine, lifestyle medicine, root-cause medicine

## Abstract

The prevalence of autism has been increasing at an alarming rate. Even accounting for the expansion of autism spectrum disorder diagnostic (ASD) criteria throughout the 1990’s, there has been an over 300% increase in ASD prevalence since the year 2000. The often debilitating personal, familial, and societal sequelae of autism are generally believed to be lifelong. However, there have been several encouraging case reports demonstrating the reversal of autism diagnoses, with a therapeutic focus on addressing the environmental and modifiable lifestyle factors believed to be largely underlying the condition. This case report describes the reversal of autism symptoms among dizygotic, female twin toddlers and provides a review of related literature describing associations between modifiable lifestyle factors, environmental exposures, and various clinical approaches to treating autism. The twins were diagnosed with Level 3 severity ASD “requiring very substantial support” at approximately 20 months of age following concerns of limited verbal and non-verbal communication, repetitive behaviors, rigidity around transitions, and extensive gastrointestinal symptoms, among other common symptoms. A parent-driven, multidisciplinary, therapeutic intervention involving a variety of licensed clinicians focusing primarily on addressing environmental and modifiable lifestyle factors was personalized to each of the twin’s symptoms, labs, and other outcome measures. Dramatic improvements were noted within several months in most domains of the twins’ symptoms, which manifested in reductions of Autism Treatment Evaluation Checklist (ATEC) scores from 76 to 32 in one of the twins and from 43 to 4 in the other twin. The improvement in symptoms and ATEC scores has remained relatively stable for six months at last assessment. While prospective studies are required, this case offers further encouraging evidence of ASD reversal through a personalized, multidisciplinary approach focusing predominantly on addressing modifiable environmental and lifestyle risk factors.

## 1. Introduction

Autism diagnoses were exceptionally rare prior to the latter decades of the twentieth century. In the early 1990s, the prevalence of autism among children in the United States was still estimated to be just 1 in 2000 [1]. Even after the autism spectrum disorders (ASD) diagnostic criteria were subsequently expanded throughout the 1990’s, CDC data suggested that only 1 in 150 children had an ASD diagnosis in 2000 [2,3,4,5,6] The most recent data from the CDC revealed that 1 in 36 children had an ASD diagnosis in 2020, representing an over 300% increase in the past twenty years utilizing similar diagnostic criteria.

The prevalence of ASD among twins has not been studied as extensively. However, limited studies among twin pairs with at least one ASD diagnosis suggest that monozygotic twins are considerably more likely to share a diagnosis (58% concordance) than dizygotic twins (21% concordance). ASD studies among twins have also revealed interesting implications regarding comparative genetic versus environmental influences on ASD diagnosis. A study of over 200 pairs of twins in whom there was an autism diagnosis revealed that environmental and lifestyle factors were a far greater contributor to ASD diagnosis than heritability, accounting for 58% versus 38% of the variance, respectively [7]. However, concern has been expressed that shared environments in ASD may be a statistical artifact of prevalence assumptions as well as potential oversampling of dizogytic concordant twin pairs, collectively leading to conclusions of greater environmental influences. Other studies, including a meta-analysis of seven studies of twins, revealed far greater heritability of between 64 and 91%. Estimates of the relative contributions of heritability and environmental factors are influenced by varying assumptions of autism prevalence, among other factors, and more research is needed to generate more precise estimates.

In the meantime, the clinical presentation of ASD is clearly varying and can present as a collection of health issues and comorbidities [8,9], not limited to neurodevelopmental, language, or social challenges [10,11,12,13,14,15]. Comorbidities include immune dysregulation [16,17,18,19,20,21,22,23], gastrointestinal issues such as diarrhea, constipation, and dysbiosis, mitochondrial dysfunction [24,25,26,27], poor detoxification [28], inflammation [29], food sensitivities, evidence of environmental toxicants [30,31,32,33,34,35,36,37,38,39,40,41,42], retained reflexes [43,44], and other structural or functional challenges.

Specific modifiable environmental and lifestyle risk factors for ASD include exposure to environmental toxicants [45,46,47], poor diet [29,48,49,50,51,52,53,54,55,56,57,58,59,60,61,62,63], disruption of the gut microbiota [64,65,66,67,68,69,70,71,72,73,74], excessive exposure to non-native electromagnetic fields (EMFs) [75,76,77,78,79,80,81,82,83,84], and accumulation of heavy metals. There are limited FDA-approved pharmacological options at present to treat ASD. Accordingly, there have been a number of non-pharmacological interventions tailored to address underlying environmental and lifestyle risk factors that have demonstrated promising, though not conclusive, improvements in ASD symptoms [85,86]. These include dietary interventions [48,62] such as gluten and casein-free [87,88,89,90,91,92,93,94,95], GAPS, a specific carbohydrate diet [48], low glutamate [96,97,98,99,100,101], and ketogenic [102,103,104,105,106,107,108,109,110,111]. While the effectiveness of a number of these dietary interventions for improving ASD symptoms has been evaluated utilizing randomized controlled designs, blinding is often challenging for such interventions, and some degree of expectation bias is possible. More studies are needed for a conclusive inference to be made with respect to the universal effectiveness of any single dietary intervention, particularly in light of the heterogeneity of both ASD etiology and presentation of symptoms. Targeted dietary supplements such as vitamin D [112,113], methylfolate [114,115], and carnitine [116,117], vitamin B_12_ and other micronutrient supplementation [51,118], mitochondrial support, or supplements thought to be relevant to a child’s functional genomic situation. Addressing other modifiable lifestyle factors and environmental interventions, such as more time in nature, a reduction in exposure to artificial light, and improving indoor air quality, have demonstrated promise. Therapeutic interventions addressing a child’s physical structure and function, such as cranial osteopathy [119,120], retained reflex integration [121,122,123], physical therapy [124,125,126], and occupational therapy, have also been associated with improved outcomes among ASD patients. As noted above for the dietary interventions, blinding was also not possible for these physical structure-oriented interventions, and some of these studies lacked a control arm, so a degree of bias exists that limits the inference with respect to their effectiveness. While reversal of ASD diagnosis is relatively rare, there have been documented cases in the literature [127,128,129] of complete recovery with multi-modal intervention. One such case achieved reversal of ASD diagnosis through a combination of dietary modifications, probiotics and micronutrient supplementation, and antimicrobials that were personalized to the child’s risk factors, clinical presentation, and a variety of labs. 

This case report, composed in alignment with the reporting guidelines of the CARE Statement [130,131], describes the reversal of the number and severity of ASD symptoms among a pair of female dizygotic twin toddlers in whom a multi-modality, non-pharmacological approach was offered by a multi-disciplinary team of clinicians.

## 2. Patient Information

**Figure 1:**
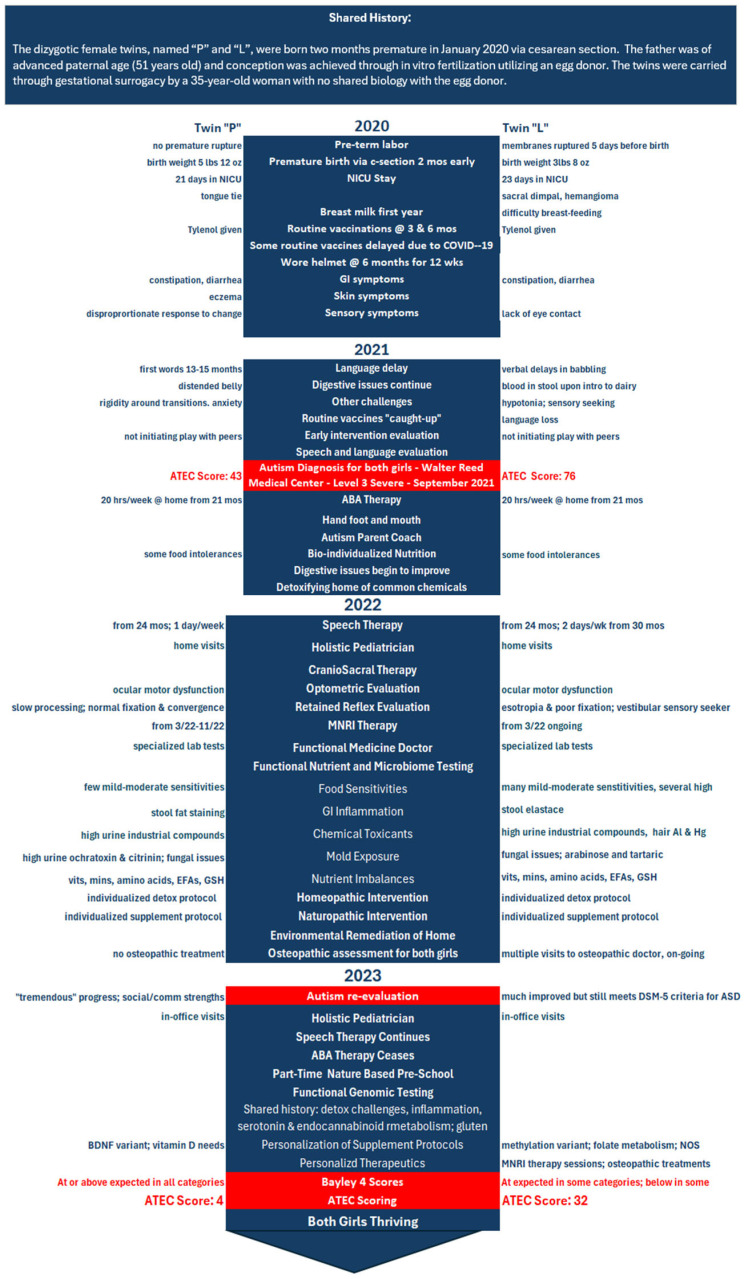
Timeline of the twins’ birth, symptoms, diagnosis, treatment strategy, and outcomes.

A timeline providing a shared history of the patients, diagnoses, treatments, changes in symptoms, and other outcomes over time is provided in Figure 1. 

### 2.1. Family History, Conception, and Gestation

The father was of advanced paternal age (51 years old), and conception was achieved through in vitro fertilization utilizing an egg donor. The fetuses were carried through gestational surrogacy by a 35-year-old woman with no shared biology with the egg donor. 

### 2.2. Birth and First Year of Life

The dizygotic female twins, named “P” and “L”, were born two months premature in January 2020 via cesarean section. L experienced a premature rupture five days before delivery. P was 5 lbs, 12 ounces at birth, and spent 21 days in the neonatal intensive care unit. L was 3 lbs, 8 ounces at birth and spent 23 days in the neonatal intensive care unit. The twins received routine vaccinations at 3 and 6 months, and then no further vaccination until 14 months of age due largely to the COVID-19 pandemic. Acetaminophen was administered prior to and following vaccines.

## 3. Primary Concerns and Symptoms of the Patients

### 3.1. Parental Concerns Prior to Autism Diagnosis—First Year of Life

In P, parents noticed stool symptoms, including occasional diarrhea, constipation, eczema, and a disproportionate response/protest to change or stimuli. In L, parents noticed stool symptoms, including constipation and diarrhea, inconsistent eye contact, babbling communication, and difficulty breast feeding. Hypotonia was observed, but motor milestones were met. There were no issues with eating or sleeping reported. Both twins received breast milk for 12 months via a combination of pumped milk from the gestational carrier and induced lactation from the mother.

### 3.2. Parental Concerns Prior to Autism Diagnosis—One Year Old until Diagnosis

At 12 months, the twins ceased drinking breast milk, and cow dairy/milk was introduced. In L, parents reported cow dairy intolerance (white stool, blood in stool, and vomiting upon exposure), constipation and diarrhea, hypotonia, lack of eye contact, sensory seeking, and language delay. In P, parents reported constipation and diarrhea, repetitive behavior/stims, rigidity around transitions, anxiety, and language delay. In March 2021, the twins received a series of “catch up” vaccines that had been delayed due to the COVID-19 pandemic. The parents noticed a worsening of some symptoms after this round of vaccinations, including significant language loss for L. In July 2021, a Strong Start Early Intervention evaluation was conducted. It was noticed that L was communicating only in single words at that time.

### 3.3. Autism Spectrum Disorders Diagnoses

In light of the concerns described above, an ASD evaluation was conducted at Walter Reed National Medical Center in September 2021. Both twins met the DSM-5 Autism Spectrum Disorders diagnosis at Walter Reed National Military Center with Level 3 severity “requiring very substantial support” at approximately 20 months of age. 

Diagnostic testing included administration of the Autism Diagnostic Observation Schedule, Second Edition (ADOS-2). The ADOS-2 provides direct observation of social and communication abilities, with activities chosen according to language and developmental level. The ADOS-2 was used qualitatively due to non-standard administration secondary to COVID-19 (i.e., use of face masks). As a result, diagnostic algorithm scores were not calculated. ADOS-2 results are described in detail below. Overall, this observation is consistent with the parent report and can be considered a valid representation of [L or P] current ability.

ADOS categories evaluated included:

- Language ability (receptively and expressively)
- Pragmatic abilities
- Articulation/phonological skills
- Voice/Fluency

The evaluation also included a summary of autism spectrum disorder symptoms that were grouped into the following four categories:

1. Language and Communication
2. Reciprocal Social Interaction
3. Play
4. Stereotyped Behaviors and Restricted Interest

A pediatrician and other specialists noted that L had no language or imaginative play, she had retained reflexes (including the Moro reflex [132,133,134]), and an eye exam showed esotropia and poor fixation. L had difficulty in social communication (she had reduced verbal and non-verbal communication), poorly modulated eye gaze, rarely gave or showed objects to others, had a flat intonation pattern, and repetitive and restrictive patterns of behaviors and interests (she demonstrated stereotyped movements, cv language and jargon, repetitive play actions, and sensory seeking behaviors). L met DSM-5 criteria for a diagnosis of autism spectrum disorder (ICD10: F84.0) with Level 3 severity (requiring very substantial support).

P showed ocular motor dysfunction with slow visual-spatial processing. P was also described as willing to explore the room but socially aloof, and demonstrating a lack of parental reference. She was showing functional pretend play but struggled to share enjoyment in joint play. P did not respond to praise or show/give any objects to her parents. Nonverbally, P’s eye contact and facial expressions were reduced. She did not use a well-formed point and struggled to follow nonverbal commands (e.g., hand out to give). P did not imitate outside of familiar tasks such as stacking blocks. She was observed engaging in repetitive play (e.g., lining up items, stacking) and sensory-driven behaviors (e.g., dose visual inspection, tactile exploration). She had some distress with transitioning away from the evaluation (e.g., was not responding to redirection). P also met DSM-5 criteria for a diagnosis of autism spectrum disorder (ICD10: F84.0) with Level 3 severity (requiring very substantial support).

### 3.4. Other Symptoms and Diagnostic Testing

Evaluation by an early intervention professional in December 2021 revealed that the twins typically played independently of each other in their home and were not initiating interactions with peers.

Multiple out-of-pocket specialty lab tests, including buccal swabs for functional genomic profile (IntellxxDNA) [135], urine organic acid [136] tests and testing for organic compounds and metabolites (Mosaic Diagnostics and Genova Diagnostics), hair analysis [137] for metals and minerals (Mosaic Diagnostics), blood analysis for nutritional (vitamin and mineral) status (Genova Diagnostics) [138] and IgG-mediated food sensitivities [139] (Mosaic Diagnostics), stool studies [140] for pathogens and GI health (Mosaic Diagnostics), and urine analysis for mycotoxins [141] (Mosaic Diagnostics) were conducted in the first year after the twin’s official autism diagnosis from March of 2022 with repeat testing in 2023. Findings of these tests in both twins included biomarkers [142,143,144,145,146,147,148,149,150,151,152,153,154,155] associated with mild gastrointestinal inflammation (one child with low elastase and the other with significant fat staining, though plentiful lactobacillus and bifidobacteria and unremarkable secretory IGAs), fatty acid imbalances (high omega 6:3 ratio), nutrient deficits (minerals tested below the 50th percentile, and both twins were relatively low in vitamins C, B_12_, and B_3_, alpha lipoic acid, glutathione) with signs of mold exposure (highly elevated urinary ochratoxin and citrinin), fungal issues (elevated arabinose for both girls, and elevated tartaric acid for L), signs of bacterial overgrowth (elevated hippuric acid for P and dihydroxyphenylpropionic acid for L), metal excretion (high aluminum in both girls), sensitivities to common foods, and urinary excretion of toxic compounds and their metabolites (some >100th percentile, glyphosate at or above 75th percentile for both twins). 

L and P were both diagnosed with ocular motor dysfunction in Spring 2022.

## 4. Therapeutic Interventions

The twins’ parents were able to work with an autism parent coach [156], who initially oriented them to the diagnosis and helped provide them with perspective and confidence. The parents were motivated to address the “total allostatic load” of stressors that is believed to underlie many chronic conditions. The total allostatic load model suggests that chronic exposure to physical, mental, or environmental stressors leads to the persistent release of primary mediators (e.g., inflammatory cytokines, cortisol) that disrupt physiological function and can lead to chronic disease [157,158]. Numerous systematic reviews have revealed that total allostatic load is associated with an increased risk of chronic disease across the lifespan, including in childhood [159,160]. The parents were exposed to this concept by reading popular books on the topic of total allostatic load and autism [158], listening to autism-focused audio materials [156] provided by their coach, and subsequently taking the Child Health Inventory for Resilience and Prevention (CHIRP) survey of the Documenting Hope Project, a comprehensive assessment of total allostatic load among children [161]. Parents who complete CHIRP receive a comprehensive report that identifies stressors contributing to the total allostatic load in the child’s health history and can be shared with health providers or used to identify areas of concern. 

In addition to completing the CHIRP survey and receiving the report, the twins’ mother also utilized additional resources through Epidemic Answers, including free webinars offered by different experts on the topic of autism and a parent forum called Healing Together [162], which provides a “road map” of steps designed to change the trajectory of complex chronic health conditions, such as autism.

Alongside Applied Behavior Analysis (ABA, which is typically recommended for new ASD diagnoses), beginning at 22 months and ending at 33 months, and speech therapy starting at 24 months, the twins’ parents implemented a rigorous diet and nutrition intervention around the time of diagnosis. They eliminated sources of glutamate in the children’s diet following the Reduced Excitatory Inflammatory Diet [163]. The twins were also put on a strictly gluten-free, casein-free diet that was low in sugar and had no exposure to artificial colors, dyes, or ultra-processed foods. An emphasis was placed on consuming organic, unprocessed, freshly prepared, and home-cooked food from local sources when possible. The family also consulted with a dietician for guidance around these dietary interventions. 

A number of dietary supplements, including omega-3 fatty acids, a multivitamin, vitamin D, carnitine, 5-methyltetrahydrofolate, and bio-individualized homeopathic remedies, were taken by both girls. A combination of labs and genomic information were utilized to inform dietary supplementation.

The family consulted a naturopathic doctor who guided them in some DNA-targeted precision medicine using the IntellxxDNA genomics tool [135]. There were some common findings, such as impaired serotonin metabolism and a recommendation that the girls be fed a diet rich in tryptophan to upregulate serotonin production, as well as consume foods rich in vitamins B_12_, B_6_, and folate. Both twins had several genetic variants, which may increase their risk of systemic inflammation. The mother was advised to feed the children foods that are high in betaine and choline, as well as to supplement with lion’s mane mushroosm and resolvins. However, each girl also had needs that were independent of each other. P had variants that may increase her need for vitamin D. L has several variants that may increase the risk of neuroinflammation, oxidative stress, and compromised detoxification. Advice was provided to support glutathione production. 

Trans-disciplinary referrals and specialized therapies helped the parents access complementary structural and functional supports for their twins. The girls had the most sessions of any intervention during the time of this reporting with an occupational therapist who focused on the specialized technique of neuro-sensory motor reflex integration [164,165,166,167] to support the integration process of primary motor reflex patterns and encourage nervous system regulation. This technique is believed to help reengage inhibited neural pathways or facilitate the activation of alternate neural pathways.

Information about the importance of addressing the potential load of environmental toxicants on the children from the autism parent coach and independent reading will lead the family to evaluate their home for air quality, mold and moisture risk, and to put supports in place that include opening windows for better cross-ventilation.

In October of 2022, a Building Biology Environmental Consultant [168] was invited to evaluate the twins’ home. The environmental consultant tested the home’s indoor air quality, evaluated possible signs of moisture intrusion, and identified other potential sources of toxicants. Air tests for mold were reported to be “very clean”. However, the family was encouraged to further evaluate several areas of the home in which a thermal imaging camera and/or a moisture meter suggested the possibility of water damage. A window in the twins’ bedroom was one area designated for further evaluation.

At the recommendation of the developmental optometrist, both girls were taken for evaluation by a cranial osteopath. The family decided to pursue osteopathic care for L and not for P. L visited an osteopath at regular intervals in 2023 and saw notable benefits, including overall disposition and communication.

## 5. Patient Outcomes

The Autism Treatment Evaluation Checklist (ATEC) is a 77-item instrument that is sensitive to change and is utilized to evaluate ASD treatment effectiveness, with lower scores indicating improvement in symptoms [169,170]. 

L’s ATEC scores improved dramatically, from 76 in March 2022 to 32 in October 2023, and then remained relatively stable at 34 in March 2024. P’s ATEC scores also improved dramatically, from 43 in March 2022 to 4 in October 2023, remaining stable at 4 in March 2024.

In addition to the twins’ improved ATEC scores, numerous other behavioral and social improvements were noted after the implementation of the interventions. L and P’s eye contact, language, and attention had all improved noticeably by Fall 2022. This was accompanied by participation in a toddler play group three days per week and ultimately attending pre-school three days per week in Fall 2023. The pediatrician noted that P had undergone “a kind of miracle”.

Clinical re-evaluation utilizing ADOS-2 at Children’s National Hospital revealed that no sensory-related behaviors were observed in P, and it was noted that she demonstrated behavioral regulation, engagement in spontaneous play, reliably socially responsive, and age-appropriate expressive and receptive language. P was still noted to have mildly repetitive speech and behavior. L was noted to have improved behavioral regulation, emerging requesting behavior (e.g., giving objects), engagement during preferred activities (e.g., peekaboo), and use of emerging phrases. L was still noted to have reduced social communication and some repetitive speech and behavior.

The twins seemed to tolerate the interventions well, as indicated by their reduced ATEC scores, pre-school success, and other outcomes. There were no adverse events noted. 

Please see Figure 1 for a timeline of the twins’ birth, symptoms, diagnosis, treatment strategy, and outcomes on the following page.

## 6. Discussion

This case revealed a reversal of the Level 3 Autism Spectrum Disorder diagnoses among dizygotic toddler twin girls that was achieved primarily through environmental and lifestyle modifications over a two-year period. The twins’ dramatic improvements and diagnosis reversal have persisted for over six months with no signs of regression. While there are numerous factors underlying these improvements, the motivation of the twins’ parents to implement environmental and lifestyle modifications was particularly notable. This included both independent, parent-led intervention as well as the guidance and therapeutic intervention of numerous practitioners (e.g., coach, physician, dietitian, occupational therapist, optometrist). Many of these practitioners commented on how the family-wide commitment to and excellent compliance with the total load-oriented interventions, the parents’ exceptional communication with practitioners, and their positive attitude all seemed to influence the positive outcomes noted in this case.

These findings and other published cases of ASD reversal are encouraging [127,128,129], as the ongoing dramatic increase in the prevalence of ASD presents challenges to the lifelong health and wellbeing of both those affected and to society more generally. For instance, the economic impact of ASD is deeply concerning and generally unappreciated at this point in time. Published projections estimate that even if the future prevalence of ASD remained unchanged over the next decade, there would be approximately 1 million new cases, thereby resulting in an additional $4 trillion of lifelong social costs in the United States. Furthermore, if the current rate of increase in prevalence continues, costs could reach nearly $15 trillion of lifelong costs by 2029 [171]. Thus, while ABA and the current standard of care can be important contributors to the management of ASD symptoms for some children [172,173,174], many with ASD do not respond, and there has been a need for personalization [175]. Investigation into other more personalized, root-cause oriented therapeutic approaches such as those described in this case is warranted.

There are several key strengths of this report. First, the meticulous documentation of diagnoses, tracking of ATEC scores over time, and detailed behavioral reports from the parents and a variety of practitioners helped rigorously establish the twins’ improvement and reversal of symptoms noted in this case. The extensive documentation allowed for the construction of a detailed timeline (Figure 1), including diagnoses, interventions, and changes in key outcomes and other symptoms over time. In addition, while there are a variety of ASD assessments in the published literature, including the Childhood Autism Rating Scale, the Autism Diagnostic Interview—Revised, and the Autism Diagnostic Observation Schedule, the ATEC is more sensitive to change with intervention than the majority of these instruments, which are generally better-suited to the diagnosis and measurement of ASD stability [170]. Another strength of this case report of dizygotic twins is the clear environmental and lifestyle influences on ASD that these findings help establish, building upon previous studies revealing the comparatively greater impact of these types of factors than genetics [7].

There are also several limitations that are worthy of mention. First, while effective at reversing ASD diagnoses, the comprehensive approach that was employed in this case may not yet be widely generalizable. For instance, the cost of the healthy lifestyle modifications and out-of-pocket costs of care of the numerous practitioners and laboratory assessments in this case would be financially prohibitive to many families. Access to healthy foods and the types of practitioners contributing to this therapeutic approach may also be limited for many families. A second limitation of any comprehensive treatment approach is the lack of clear evidence of the isolated effects of each of the modalities that were employed. However, it has become increasingly clear that ASD treatment is not one-size-fits-all and that personalized, multi-modality treatment approaches to help address the total load of stressors are likely required to achieve optimal outcomes.

## 7. Conclusions

The dramatic improvement and reversal of ASD diagnoses among these fraternal twins demonstrates the potential of a comprehensive treatment approach including both conventional therapies (e.g., ABA) and a wide variety of environmental and lifestyle modifications facilitated by a multi-disciplinary team of practitioners addressing the total load of stressors of modern living. The commitment and leadership of well-informed parents or guardians is an essential component of the effective personalization that appears necessary for the feasibility of such improvements. Future prospective studies are warranted to confirm these findings.

## 8. Patient Perspective

“Having fraternal twin daughters diagnosed with Autism Spectrum Disorder at 20 months has given us a profound appreciation of the highly individual presentation of Autism. Despite sharing similar genes and identical conception, gestation, birth experience, and post-natal factors—as well as benefitting from consistent nurture, home environment and family dynamics—each daughter presented an ASD diagnosis entirely uniquely. Early in our navigation, we adopted a ‘total load’ theory, understanding that there was not one ’single’ factor that catalyzed their diagnosis but the combined assault of many injuries. In accepting the very complex presentation of ASD, we also understood that there would be no singular ‘cure’ for it either. Conventional statistics have stacked the odds against the ability to recover a child from an ASD diagnosis. Our approach was therefore focused on following a nonconventional, holistic understanding of each daughter’s bio individual needs, exploring root cause and designing customized support. We began by choosing to pursue functional, integrative support centered around the foundational principles of diet, environment, and lifestyle. We vetted therapies that would deliver support in a naturalistic setting—primarily our home. We chose practitioners who were aligned in our belief in our daughters’ intrinsic ability to heal given the right support. We assembled a team that welcomed our engagement and worked cooperatively with each other. We committed to being highly involved in all the interventions we explored, educating ourselves and advocating for what we felt was best for our children. Finally, we were strategic in focusing on sequence, using labs and other evaluation tools to measure progress, and harnessing the power of innovation to map genes and address cellular health. We explored modalities both new and old, while operating from a place of patience and curiosity. Most importantly, in our experience as parents has been the desire to create and maintain a profound and loving bond with each of our daughters—and to remain parents, not practitioners. Through this approach, we have witnessed the radical recovery of one daughter—who presents today as a joyful, engaging, spirited, extremely bright 4-year-old. We remain steadfast in our support for our other daughter whose progress has also consistently amazed us and has reminded us that recovery is possible at each person’s individual pace”.

## Data Availability

The data presented in this case report are available from the corresponding author upon reasonable request. The data are not publicly available to preserve family privacy.
